# MMP-9/BDNF ratio predicts more severe COVID-19 outcomes

**DOI:** 10.7150/ijms.75337

**Published:** 2022-10-24

**Authors:** Goran Savic, Ivana Stevanovic, Dusan Mihajlovic, Milena Jurisevic, Nevena Gajovic, Ivan Jovanovic, Milica Ninkovic

**Affiliations:** 1 Medical Faculty of the Military Medical Academy, University of Defence, Crnotravska 17, 11000 Belgrade, Serbia.; 2 Institute of Medical Research, Military Medical Academy, Crnotravska 17, Belgrade, Serbia.; 3 Department of Pharmacy, Faculty of Medical Sciences, University of Kragujevac, Svetozara Markovica 69, 34000 Kragujevac, Serbia.; 4 Centre for Molecular Medicine and Stem Cell Research, Faculty of Medical Sciences, University of Kragujevac, Svetozara Markovica 69, 34000 Kragujevac, Serbia.

**Keywords:** COVID-19, MMP-9, BDNF, VEGF A, MMP-8

## Abstract

COVID-19 clinically manifests from asymptomatic to the critical range. Immune response provokes the pro-inflammatory interactions, which lead to the cytokines, reactive oxygen/nitrogen species, peptidases, and arachidonic acid metabolites enlargement and activation of coagulation components. Matrix metalloproteinases (MMPs) contribute to tissue destruction in the development of COVID-19. Due to the endothelial, systemic course of the disease, VEGF A participates actively in COVID-19 development, while neurotrophic and metabolic effects of BDNF recommends for the prediction of complications in COVID-19 patients. Searching for a marker that would improve and simplify the ranking in COVID-19, the study intended to evaluate the relationship of MMP-9 with VEGF A, BDNF, and MMP-8 with the COVID-19 severity. Upon admission to the hospital and before the therapy administration, 77 patients were classified into a mild, moderate, severe, or critical group. Due to the inflammatory stage in COVID-19, a comparison between groups showed related differences in leukocytes, neutrophils, lymphocytes, and platelets counts as anticipated. Only in seriously ill patients, there is a significant increase in the serum concentration of MMP-9, MMP-8, and VEGF A, while BDNF values did not show significant variations between groups. However, all those parameters positively correlated with each other. The ratio of MMP-9/BDNF markedly decreased in the severe and critically patients compared to the mild group. Testing the capability of this ratio to predict the COVID-19 stage by ROC curves, we found the MMP-9/BDNF could be a suitable marker for differentiating stages I/II (AUC 0.7597), stage I/III (AUC 0.9011), and stage I/IV (AUC 0.7727). Presented data describe for the first time the high-level systemic MMP-9/BDNF ratio in patients with COVID-19. This parameter could contribute to a more precise determination of the phase of the disease.

## Introduction

The appearance and spread of coronavirus disease (COVID-19) from 2019 to nowadays have posed unforeseen pandemic challenges 1,2. COVID-19 clinically manifests from asymptomatic to the critical range, with dominantly respiratory insufficiency but also thromboembolic complications in different organs and tissues 3. Using the angiotensin-converting enzyme 2 (ACE2) virus enters the cells, sometimes potently stimulating the development of systemic inflammatory disease. 4. Immune-inflammatory processes, activated by uncontrolled/suppressed immune reactions, result in overproduction of cytokines, reactive oxygen/nitrogen species, peptidases, arachidonic acid metabolites as well as activation of coagulation components 5. Further, their synergistic actions can promote the course of the disease towards deterioration, developing acute respiratory distress syndrome, sepsis, septic shock, and death 6.

Matrix metalloproteinases (MMPs) are prominent mediators of tissue destruction and immune activity, whose importance has also been noted in the development of COVID-19 7. Increased activities of matrix metalloproteinase 9 (MMP-9), as well as matrix metalloproteinase 8 (MMP-8), were reported in patients with severe form of COVID-19 8,9. MMP-9 is a zinc-metalloproteinase (collagenase type IV; gelatinase B) that could alternate for primed neutrophils, while MMP-8 (neutrophil collagenase) degrades type I, II and III collagen of extracellular matrix. It is shown that MMP-9 could be a suitable marker of lung injury and systemic outcome in COVID-19 10. MMP-9 activates signalling molecules such as cytokines or chemokines thus contributing to the development of “cytokine storm” in COVID-19. In progressive pathological processes, it aggravates activity during degradation and regeneration of the extracellular matrix, with a particularly potent influence on vascular remodelling 11. Furthermore, by cleaving the chains of many regulatory pro-peptide molecules, MMP-9 facilitates the release of operative, mature forms of peptides. That also occurs during the transformation of pro-peptides into the brain-derived neurotrophic factor (BDNF) or vascular endothelial growth factor A (VEGF A) 12,4. Vascular endothelium contributes to thrombosis/thrombolysis, vessel wall-platelet/leukocyte interaction, and vasomotion control. The functionality of the endothelium is supervised by VEGF A, which participates actively in COVID-19 development 13. Besides the nervous system, BDNF is abundant in peripheral blood, derived predominately from lymphocytes and monocytes. Azoulay and colleagues advised that serum BDNF could be convenient as a disease severity biomarker 14. Neuromodulator, neurotrophic, metabolic and systemic effects of BDNF could be suitable for predicting severe complications in COVID-19 patients 15,16.

Searching for a marker that could improve and simplify the estimation of COVID-19 progress and considering the predictive potency of MMP-9, the study intended to evaluate the relationship of MMP-9 with VEGF A, BDNF, and MMP-8, concerning the development, severity of disease and outcomes of COVID-19.

## Materials and methods

### Patients and data collections

The investigation was conducted from May to December 2020 as cross-sectional study which included hospitalized patients with confirmed COVID-19 by real-time reverse transcription-PCR (RT-PCR) analysis. To assess the development and severity of the disease, a total of 77 hospitalized patients were categorized by an experienced clinician into one of four groups:

- I - Mild, n=22; revealing patients with fever 37-38 °C, sore throat, headache, nausea, vomiting, fatigue, myalgia, less often dry cough, SaO_2_<92-100% and pO_2_ 8.5 KPa (64 mm Hg) -13.3 KPa (100 mm Hg) with normal respiratory noise and CXR.
- II - Moderate, n=16; revealing patients with fever of 38-39 °C, frequent dry cough, sore throat, headache, nausea, vomiting, fatigue, myalgia, SaO_2_ 83-91% and pO_2_ 7.1 KPa (53 mm Hg) - 8.4 KPa (63 mm Hg), reduced/sharpened respiratory noise in the inferior lungs' portions with interstitial condensing or focal zones of consolidation in CXR.
- III - Severe, n=20; manifesting high fever (39-39.5 °C), fatigue, myalgia, headache, frequent dry irritating cough and chest pain, dyspnoea, SaO_2_ 75-82% and pO_2_ 5.6 (42 mm Hg) -7 KPa (52 mm Hg), auscultatory reduced/ silenced respiratory noise, on CXR multifocal zones of consolidation, necessitated of high-frequency ventilation (HFV) or non-invasive mechanical ventilation (Non-Invasive Ventilation, NIV).
- IV - Critically, n=19; revealing fever of more than 39.5 °C, dyspnoea, fatigue, myalgia, headache, frequent dry irritating cough and chest pain, fatigue, SaO_2_ 68-74 % and pO_2_ 4.4 (31 mm Hg) -5.5 KPa (41 mm Hg), auscultatory silenced respiratory sound, in CXR- diffuse alveolar breakdown and ARDS which requires invasive mechanical ventilation.

The patients who took medication for any of the following drugs: antibiotics, corticosteroids, statins, immunosuppressants, aminosalicylates or any of the biological drugs less than two months before being diagnosed with COVID-19, were excluded from the study.

### Measurement of biochemical parameters

At the time of admission to the hospital and before applying any therapy, blood samples were taken by venous puncture from all patients involved in the study. The blood samples were separated into four tubes- for blood cell counting, biochemical parameters, D-dimer, and immunoassay analysis. Also, all patients underwent arterial blood gas analysis- SaO_2_, pO_2_ and pCO_2_, using ion-selective electrodes on the automaton. Biochemical parameters that were determined included glucose, urea, creatinine, total bilirubin (BILT), direct bilirubin (BILD), aspartate aminotransferase (AST), alanine aminotransferase (ALT), albumin, lactate dehydrogenase (LDH), creatinine kinase (CK), C-reactive protein (CRP), procalcitonin (PCT), ferritin, iron, (Fe), potassium (K+) and sodium (Na+) (Beckman Coulter AU 400 Unicel DXC 800 Synchron Clinical System).

Serum samples were separated from collected blood and stored at -80 °C before the analysis. Commercially available ELISA essays were used for measurement of MMP-9, MMP-8, BDNF and VEGF A in serum samples according to the manufacturer′s instructions (MMP-9 and MMP-8-R&D Systems, BDNF- Elabscience Biotechnology Inc., VEGF A- Invitrogen Thermo Fischer Scientific). Cytokines-TNF-α, IL-1β, IL-6, TGF-β, IL-4, IL-10, and IL-17 were determined by commercial ELISA tests following the instructions of the manufacturer (R&D Systems, Minneapolis, Minn, USA).

### Statistical Analysis

Results are presented as counts or percentages- medians or means and standard deviations (SD). Data were analysed by Statistical Analysis Software IBM SPSS (version 23.0). Implemented significance tests were the Chi-square test, Kruskal-Wallis test and Mann-Whitney U test, and one-way ANOVA followed by Bonferroni post hoc testing. Pearson's or Spearman's correlation considered the potential association among variables. Receiver Operating Characteristic (ROC) curve analysis was used to measure optimal cut-off values of the MMP9/BDNF ratio to estimate COVID-19 severity. The statistical significance was set at *p*<0.05.

## Results

Based on the clinical assessment, the selected 77 patients have been classified into one of four groups upon admission to the hospital (**Table 1**). The oldest patients had the most severe clinical manifestation of the disease. In contrast to the mildest form (I), a marked predominance of men to women is registered among the hospitalized patients from the second stage (II) of the disease.

Clinical symptoms and signs of fever, dry cough and fatigue did not differ significantly between the groups, regardless of whether it was the mildest form of the disease or whether the disorder reached a critical stage. However, dyspnoea was more pronounced in critically ill patients compared to mildly ill patients (p<0.01). Myalgia, anosmia, headache, chest pain and sore throat did not show significant difference between four groups of hospitalized patients. In general, normal auscultatory findings did not register in the severe group of patients (p<0.05). Although obtaining sharpened respiratory sound, diffusely audible cracks, and whistling did not significantly differ among classified groups, while attenuated breathing sound was increased in patients in stage IV (p<0.05).

Concerning the results of the blood parameters (**Table 2**), severe patients (group III) had increased white blood cell count compared to the mild- I group (p<0.01), as well as the moderate- II group (p<0.05). In contrast to this increment, critically ill patients (IV group) had significantly decreased number of white blood cells compared to group III (p<0.05). In both severe and critical patients, the percentage of the neutrophil count was elevated compared to mild patients (p<0.05). Similarly, the neutrophil count was increased in the III group compared to the II group (p<0.05). With the progression of the disease, the percentages of lymphocytes and monocytes progressively decreased, reaching significance in the severely ill compared to mildly (p<0.01; p<0.05) and moderately group (p<0.01; p<0.05). The number of monocytes in patients in stage IV was significantly higher compared to the stage III patients (p<0.05). With the development of the disease, a significant rise in the number of platelets was observed in group III compared to group II (p<0.05).

From the mild to the critical stage of the disease, there was a progressive decline of pO_2_ and SaO_2_ in arterial blood.

Increased levels of blood BILT (p<0.05), BILD (p<0.01), AST (p<0.01), ALT (p<0.05) and ferritin (p<0.05) were detected in moderate ill patients in comparison to the mild group. In severely ill patients, increased glucose (p<0.01), urea (p<0.05), BILT (p<0.001), BILD (p<0.001), AST (p<0.05), LDH (p<0.01), D dimer (p<0.05) and PCT (p<0.05) were found compared to mildly ill patients, while the concentration of albumin and Fe decreased (p<0.05). The concentration of albumin in this group was also depleted compared with moderately (II) ill patients (p<0.01), while LDH and K^+^ increased (p<0.05). Critically ill patients, compared with mild patients exposed increased glucose (p<0.01), urea (p<0.001), creatinine (p<0.001), BILT (p<0.01), BILD (p<0.001), AST (p<0.001), ALT (p<0.01), LDH (p<0.001), CK (p<0.05), D dimer (p<0.05), CRP (p<0.001), PCT (p<0.05), ferritin (p<0.001) and IL-6 (p<0.001), while albumin and Fe decreased. In this group of patients, urea, LDH, CRP (p<0.01) and IL 6 (p<0.001) were also increased compared to group II, while albumin and iron have reduced (p<0.001). An increase in IL-6 and a reduction in iron in critically ill patients were prominent compared to the remaining three groups of patients (p<0.001).

Only in seriously ill patients (group III), there is a significant increase in the serum concentration of MMP-9, MMP-8, and VEGF A, while serum BDNF values between groups do not show significant variations (**Table 2**).

All four serum parameters- MMP-9, MMP-8, BDNF and VEGF A positively correlated with each other (p<0.01) (**Table 3**). They all positively correlated with cytokine TGF-β and cytokine ratio IL-10/IL-17 (cytokine data available in supplementary material). Negative correlation was detected between BDNF and anti-inflammatory parameters IL-10, IL-10/IL1, IL-10/IL-6, IL-10/IL-17, and IL-10/TNF-α. The same trend of negative correlation was noted between VEGF A and IL-10, IL-10/IL-6, IL-10/IL-17. Additionally, VEGF A positively correlated with IL-17. Negative correlation was revealed between MMP-8 and ratio IL-10/IL-6 and IL-10/TNF-α.

**Figure 1:**
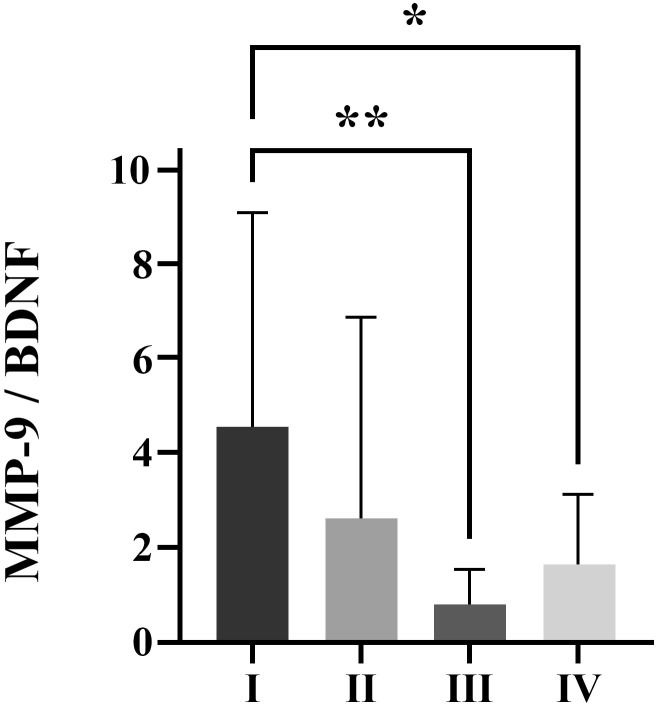
MMP-9/BDNF ratio in COVID-19 patients classified by disease severity scores into I (mild), II (moderate), III (severe) and IV (critical) groups of patients. ^*^ p<0.05; ^**^ p < 0.01 statistical significance compared to I group. A one-way ANOVA revealed that there was a statistically significant difference for the MMP-9/BDNF ratio between the groups (F(3, 66) = [4.741], p = 0.0002). MMP-9 matrix metalloproteinase-9; BDNF brain-derived neurotrophic factor.

Significant decrement of MMP-9/BDNF ratio was measured in severe (p<0.01) and critical patients (p<0.05) compared to mild patients (**Figure 1**).

**Figure 2:**
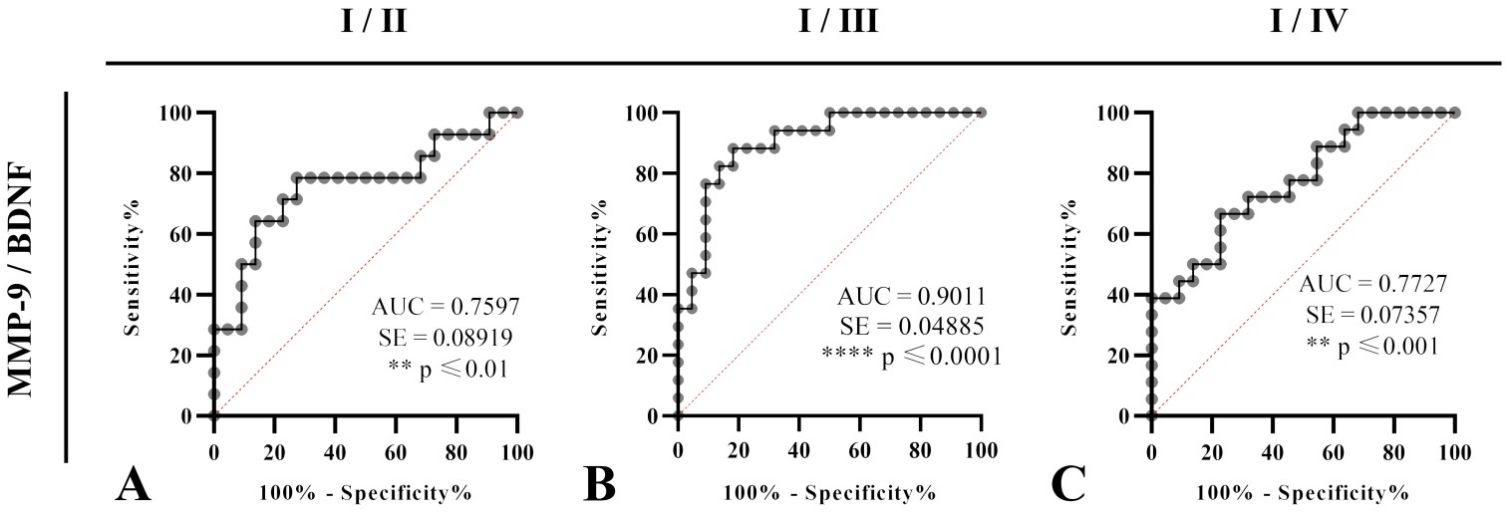
**A-C.** Receiver Operating Characteristics (ROC) curves were analysed to estimate the capability of serum MMP-9/BDNF ratio to predict the COVID-19 severity staging and the progression assessment-stages I/II (A), I/III (B), and I/IV (C). Percentages of sensitivity and specificity are displayed on each figure. A measurement of separability was expressed by the area under the curve (AUC), with a 95% confidence interval. Statistical significance presented by p. Ratio of AUC>0.7 is suitable for a COVID-19 outcomes prediction.

Test of ROC curve revealed that MMP-9/BDNF ratio can be treated as suitable marker for the disease stage assessment, from I (mild) stage upon II (moderate) (sensitivity 78.57%, specificity 72.73%; cut off: 564.8), stage III (severe) (sensitivity 88.24%, specificity 81.82%; cut off: 889.6) and IV (sensitivity 66.67%, specificity 77.27%; cut off: 598.5) in COVID-19 patients (**Figure 2**).

Correlation analysis of MMP-9/BDNF ratio with parameters of interest for the clinical assessment of the disease severity (**Table 4**) revealed its negative correlation with IL-10/IL-17 ratio in moderately ill patients (p<0.05) and highly significant positive correlation with MMP-8 in mild (I), moderate (II) and IV (critical) groups (p<0.001).

## Discussion

The study investigated the relationships between clinical, biochemical parameters and active tissue mediators (MMP-9, MMP-8, BDNF, and VEGF A), that predominate during COVID-19, depending on the stage of the disease. The limitations of this cross-section study were a single timepoint evaluation and the small number of COVID-19 patients. Without the influence of therapy and after excluding many patients from the study, patients were classified according to the previously mentioned criteria for admission to the hospital.

The oldest patients had the most prominent clinical manifestations (Table 1). Compared to the mild group, a marked predominance of men to women is registered in other three groups of patients. That is consistent with earlier published data on worsened disease outcomes of older males 17. Percentage of fever, dry cough, fatigue, myalgia, anosmia, headache, chest pain and sore throat did not differ among the groups while the dyspnoea and attenuated breathing sound were more pronounced in critically ill patients. From the mild to the critical stage of the disease, progressive decline of pO_2_ and SaO_2_ was detected in arterial blood (Table 2).

Due to the inflammatory stage in COVID-19, a comparison between groups showed related differences in leukocytes, neutrophils, lymphocytes, and platelet counts as anticipated. The severe group had a rise in white blood cell count compared to the mild and moderate group but then depleted in the critically ill patients. The fact that the oldest patients were in the critical group could considerably influence the obtained results and thus modify the monitored parameters. In both severe and critical patients, the percentage of the neutrophil count elevated while the lymphocytes and monocytes percentage progressively depleted. Platelet counts generally did not deviate significantly from referent values in any group, but in the group of severely ill patients, it increased compared to moderately ill patients. Concerning trends follow published data 18. The resulting interaction of neutrophils with activated platelets promotes the release of neutrophil-extracellular traps (NETs), that further capture granule-derived enzymes, cytoplasmic proteins, DNA, and citrullinated histones 19. NETs are primarily created to restrict inflammation. Unfortunately, by changing their framework, they can support inflammation and thrombosis. This scenario can happen when MMP-9 released from tertiary granules in activated neutrophils, attacks NETs and causes endothelial dysfunction 20.

Increment of BILT, BILD, AST, ALT, and ferritin values were detected in moderate ill group of patients. Glucose, urea, BILT, BILD, AST, LDH, D dimer, and PCT were higher in severe group, while critically ill patients, in addition to these parameters, had increased levels of creatinine, ALT, CK, CRP, ferritin and IL-6. The concentration of albumin and Fe decreased during the disease progression. An increase in IL-6 and a reduction in iron in critically ill patients were prominent compared to the remaining three groups of patients. IL-6 and its downstream target CRP reflect cytokine storm in the critical stage, thus, serving as useful indicators of respiratory failure and inferior prognosis 21. The resulting trend of change is per published data, indicating systemic damage in different tissues and organs, expressing the potential to cause a poor COVID-19 outcome 22,23. Overwhelmed response with raised levels of numerous cytokines could induce unselective tissue damage, that further can be harmful for patient 24. High levels of PCT found in severely and critically ill patients can indicate the probable occurrence of multiple tissue changes in response to cytokine overproduction, as increased PCT is not a specific marker of viral infection 25.

Considering the proven systemic effects of COVID-19 infection, tissue remodelling is a process by which the infection spreads and worsens. MMPs released by the action of tissue inhibitor of MMPs/nitric oxide, affects endothelium, which leads to increased extravasation and the development of extracellular oedema 26. The upregulated MMP-9 gene in COVID-19 patients, together with increased level of MMP-9 are directly proportional to a risk of respiratory failure 27. Our results revealed higher values of MMP-9 in the group of seriously ill patients (Table 2) that is in line with published data on upregulated MMP-9 28, but not with data on an early increment of circulating MMP-9 in COVID-19 patients with respiratory failure 29. Increased MMP-9 in severe COVID-19 patients, possibly originated from migrated neutrophils, degrades the alveolar-capillary barrier, attracts inflammatory cells, and damages lung parenchyma 30. Degradation of extracellular collagen, elastin and connexins by MMP-9 are emphasized with rapid turnover of stiffer collagen, that could be an inducer of fibrosis and numerous complications in COVID-19. Comprehensive analysis of the cytokine profiles revealed a predomination of pro-inflammatory cytokines IL-1β, TNF-α, IL-6 over IL-4 and IL-10 (supplementary table S1) during disease progression. Recent data regarding the ratio of the IL-6 and IL-10 suggested that serum cytokines may be suitable for prediction of severity of COVID-19 31. These observations are based on increased levels of multiple cytokines, most prominently IL-6, a cytokine with a resilient pro-inflammatory effect 38. The data proposed that predictive values of IL-10 and IL-6 should be preferentially evaluated for the early diagnosis of severe forms of the disease 32. Therewith, values of MMP-9 strongly positively correlated with transforming growth factor-β (TGF-β) and negatively correlated with the anti-inflammatory ratio IL-10/IL-17 and IL-10/TNF-α (Table 3). This is consistent with MMP-9-related proteolytic cleavage and consecutive activation of latent TGF-β, which could promote epithelial to mesenchymal signal transition and fibrogenesis 33,34.

MMP-9 also positively correlated with MMP-8, VEGF A, and BDNF (Table 2). The primary function of MMP-8 is the zinc-calcium-dependent endopeptidase activity that can degrade all components of the extracellular matrix (type I, II and III collagens). In COVID-19, the MMP-8 gene is seen as one of the main regulators of neutrophil-driven repair of injured lungs, as the MMP-8 is released from neutrophil secondary granules, performing the role of neutrophil chemoattractant 35,36. In investigated COVID-19 patients, MMP-8 displayed a strong positive correlation with TGF-β and negative correlation with rations IL-10/IL-6, IL-10/IL-17, and IL-10/TNF-α, thus suggesting predomination of anti-inflammatory cytokines and complementing the pro-inflammatory effects of MMP-9 and IL-6 37.

Endothelial and glycocalyx injury, together with capillary impairment, as an outstanding event in COVID-19, are followed by elevated VEGF A, a potent permeability-inducing factor related to disease severity 38. In our study, VEGF A reached significantly elevated values in severe staged patients, like MMP-9 and MMP-8 (Table 2). Linking the effects of MMP-9 and MMP-8, VEGF A displayed a potent positive correlation with IL-17, TNF-α, and TGF-β and a negative correlation with IL-10 and IL-10/IL-6 and IL-10/IL-17 ratios (Table 3). VEGF-A upregulation in severely ill patients could be induced by impaired tissue perfusion and compromised oxygen delivery, as a result of the endothelial disruption and cytokines-mediated inflammatory activation 39.

Among the defined COVID-19 groups, no changes in BDNF were observed not even in the critically ill group (Table 2). This differs in part from the findings of other authors, as they have found elevated levels of BDNF with the development of lung injury 40. Regardless of the unchanged values of BDNF in different phases of COVID-19, we found a positive correlation between BDNF and MMP-9, MMP-8, and VEGF A, as well as a strong negative correlation of BDNF with IL-10 and cytokines ratios IL-10/IL-1, IL-10/IL-6, IL-10/IL-17, and IL-10/TNF-α (Table 3). Although implemented studies have shown that plasma IL-6 and IL-10 could be used as factors to predict the progression of COVID-19, their values could not be used separately to distinguish COVID-19 stage groups. Close to VEGF A, BDNF correlated positively with TGF-β, indicating a tissue repair and the reconstruction of fibrotic tissue following lung damage 41. Further data confirmed the interconnection of BDNF and MMP-9 influences. Through signalling events, BDNF stimulates the gene transcription for MMP-9, while MMP-9, with its proteolytic activity, stimulates the formation of the mature form of BDNF, so prospectively amplifies its synthesis 42. BDNF can bind to the tyrosine kinase receptor B (TrkB) and activate the extracellular signal-regulated kinase 1/2 (ERK-1/2). ERK1/2 is aberrantly expressed in ischemia-reperfusion injury, which is expected to happen and enlarge during COVID-19 evolution. Depending on the phase of ischemic injury and tissue zone, this pathway could stimulate distinct outcomes- from inhibition of apoptosis to initiation of necrosis 43.

Considering the interrelation of MMP-9 and BDNF, to our knowledge, for the first time, we analysed MMP-9/BDNF ratio in the COVID-19, in order to link these mediators with stages of disease development. Although no change in BDNF was observed, the MMP-9/BDNF ratio was significantly depleted in severely (p<0.01), as well as in critically (p<0.05) ill patients, compared to the mild group (Figure 1). Testing the capability of this ratio to predict the COVID-19 stage by ROC curves (Figure 2), we found the MMP-9/BDNF could be a suitable marker for differentiating stages I/II (AUC 0.7597), stage I/III (AUC 0.9011), and stage I/IV (AUC 0.7727). The correlation of the MMP-9/BDNF ratio did not show significant relationships with generally known parameters used in the assessment of the COVID-19 (Table 4). An exception was a negative correlation between MMP-9/BDNF with IL-10/IL-17 in moderately ill and a highly positive correlation of this ratio with MMP-8 in mild, moderate, and critically COVID-19 groups. During acute lung injury, it has been found that MMP8 participates actively 44. Underlain by its proteolytic activity on matrix and non-matrix proteins besides the neutrophil, MMP8 could modulate the function of various immune and non-immune cell types. Endothelial cells are mostly susceptible, both from the aspect of damage during the systemic course of the COVID-19 and from the data of significant secretory activity that modulates the development of the disease 45.

Presented data describe for the first time the high-level systemic MMP-9/BDNF ratio in patients with COVID 19, which could contribute to a more precise determination of the phase of the disease. The detailed mechanism and relevance of its change during the development of COVID-19 have yet to be clarified.

## Supplementary Material

Supplementary table.

## Figures and Tables

**Table 1:** Demographic, clinical, and laboratory parameters of COVID-19 patients ranked by disease severity in the I (mild), II (moderate), III (severe) and IV (critical) COVID-19 group

	I (mild), n=22	II (moderate), n=16	III (severe), n=20	IV (critical), n=19	*p*
**Age mean±SE**	51.23±4.19	57.88±3.45	60.11±2.57	67.42±1.76^**#^	*H(3)=8.728; **p=0.033***
**Sex**					
Female	11(50.0%)	6(37.5%)	2(10.0%)	5(26.3%)	*χ2 (3)=8.321; **p=0.040***
Male	11 (50.0%)	10(62.5%)	18(90.0%)	14(73.7%)	
**Clinical symptoms**					
Fever	17(77.3%)	16(100%)	17(85.0%)	14(73.7%)	*χ2 (3)=5.041; p=0.169*
Dry cough	17(77.3%)	11(68.8%)	12(60.0%)	16(84.2%)	*χ2 (3)=3.253; p=0.354*
Fatigue	9(40.9%)	12(75.0%)	12(60.0%)	12(63.2%)	*χ2 (3)=4.785; p=0.188*
Dyspnoea	4(18.2%)	6(37.5%)	9(45.0%)	13(68.4%) ^**^	*χ2(3)=10.801; **p=0.013***
Nausea and vomiting	11(50%)	5(31.3%)	11(55.0%)	6(31.6%)	*χ2(3)=3.530; p=0.317*
Myalgia	1(4.5%)	5(31.3%)	3(15.0%)	3(15.8%)	*χ2 (3)=5.028; p=0.170*
Anosmia	2(9.1%)	3(18.8%)	3(15.0%)	1(5.3%)	*χ2 (3)=1.889; p=0.596*
Headache	2(9.1%)	2(12.5%)	2(10.0%)	1(5.3%)	*χ2 (3)=0.582; p=0.901*
Chest pain	1(4.5%)	2(12.5%)	1(5.0%)	4(21.1%)	*χ2 (3)=3.828; p=0.281*
Sore throat	0(0%)	1(6.3%)	1(5.0%)	0(0%)	*χ2 (3)=2.393; p=0.495*
**Auscultatory results**					
Normal	8(36.4%)	1(6.3%)	1(5.0%)^*^	0(0%)	*χ2(3)=15.246; **p=0.002***
Attenuated breathing sound	7(31.8%)	9(56.3%)	12(60.0%)	13(68.4%) ^*^	*χ2(3)=15.171; **p=0.002***
Sharpened respiratory sound	1(4.5%)	3(18.8%)	1 (5.0%)	3(15.8%)	*χ2 (3)=3.227; p=0.358*
Audible cracks diffusely	10(45.5%)	6(37.5%)	13(65.0%)	11(57.9%)	*χ2 (3)=3.344; p=0.342*
Audible whistling	1(4.5%)	0(0%)	0(0%)	1(5.3%)	*χ2 (3)=1.824; p=0.610*

Kruskal-Wallis test (H(df)= Kruskal-Wallis test statistics) was performed followed by post-hoc test using the Mann-Whitney U test presented as ^*^ p<0.05; ^**^ p < 0.01 statistical significance compared to I group; ^#^ p<0.05; ^##^ p < 0.01 statistical significance compared to II group or Chi-squared test (*χ2*(df)= Pearson Chi-Square test statistics).

**Table 2:** Laboratory parameters of COVID-19 patients (n=77) ranked by disease severity in the I (mild), II (moderate), III (severe) and IV (critical) group

	I (mild), n=22	II (moderate), n=16	III (severe), n=20	IV (critical), n=19	*p*
Blood analysis					
White blood cell count, x10^9^/L	6.38±0.56	7.89±1.52	9.64±0.87^**#^	5.73±0.75^£^	*H(3)=13.345; **p=0.004***
Neutrophil count %	69.19±3.09	75.16±2.35	79.48±4.49^*#^	76.99±2.75^*^	*H(3)=10.779; **p=0.013***
Lymphocyte count %	22.45±2.73	16.86±1.90	10.76±1.60^**##^	11.7±1.84^*^	*H(3)=15.923; **p=0.001***
Monocyte count %	10.06±0.11	7.41±0. 71	5.03±0.46^*#^	9.9±1.86^£^	*H(3)=12.491; **p=0.006***
Erythrocyte count, x10^12^/L	4.38±0.12	4.56±0.12	4.62±0.09	4.37±0.13	*H(3)=4.027; p=0.259*
Thrombocyte count, x10^9^/L	230.09±13.85	205.63±31.28	266.84±20.58^#^	209.42±29.85	*H(3)=7.188; **p=0.049***
Haemoglobin g/L	125.5±3.32	134.62±3.76	132.42±2.56	127.63±13.57	*H(3)=4.467; p=0.215*
**Arterial blood gasses**					
pO_2_ kPa	10.38±0.48	8.30±0.29^**^	7.52±0.24^*** #^	6.44±0.26^*** ###^	*H(3)=38.133; **p=0.001***
pCO_2_ kPa	4.57±0.15	4.61±0.17	4.45±0.16	4.89±0.18	*H(3)=3.273; p=0.351*
SaO_2_ %	95.59±0.58	92.81±0.61	90.53±0.75^***^	83.26±1.89^*** ###^	*H(3)=41.617; **p=0.001***
pH	7.46±0.01	7.49±0.01^**^	7.31±0.15	7.46±0.01^#^	*H(3)=8.105; **p=0.044***
**Biochemical analysis**					
Glucose mmol/L	6.42±0.48	8.07±1.06	9.78±1.09^**^	9.33±0.99^**^	*H(3)=14.231; **p=0.003***
Urea mmol/L	5.75±0.76	5.99±0.70	8.72±1.24^*^	10.87±1.27^*** ##^	*H(3)=15.977; **p=0.001***
Creatinine umol/L	110.41±31.57	93.43±5.28	97.36±9.34	145.84±25.77^*** £^	*H(3)=12.547; **p=0.006***
BILT umol/L	8.41±0.49	12.32±1.74^*^	12.05±0.75^***^	11.05±0.65^**^	*H(3)=14.930; **p=0.002***
BILD umol/L	1.88±0.15	3.64±0.53^**^	3.91±0.37^***^	3.83±0.42^***^	*H(3)=21.753; **p=0.001***
AST U/L	34.23±3.69	56.5±7.85^**^	46.10±4.13^*^	59.1±5.81^***^	*H(3)=16.518; **p=0.001***
ALT U/L	37.27±7.52	56.75±7.85^*^	48.53±7.10	55.39±8.52^**^	*H(3)=8.955; **p=0.030***
Albumin g/L	37.72±2.04	37.40±0.99	33.42±0.87^* ##^	31.72±0.94^** ###^	*H(3)=16.519; **p=0.001***
LDH U/L	521.20±42.08	575.23±67.34	862.81±80.54^** #^	1140.19±133.30^*** ###^	*H(3)=22.904; **p=0.001***
CK U/L	150.09±37.05	204.75±51.25	108.39±19.99	491.68±193.65^* ££^	*H(3)=9.946; **p=0.019***
D dimer ug/mL	1.45±0.69	1.23±0.34	2.45±0.95^*^	1.94±0.42^*^	*H(3)=9.424; **p=0.024***
CRP mg/L	48.26±10.58	89.52±22.25	131.86±18.26^***^	169.43±19.23^*** ##^	*H(3)=25.687; **p=0.001***
PCT ng/mL	0.12±0.04	0.12±0.03	0.33±0.13^*^	0.33±0.09^*^	*H(3)=9.356; **p=0.025***
K^+^ mmol/L	3.87±0.09	3.48±0.12^**^	3.88±0.08^#^	3.65±0.13	*H(3)=9.114; **p=0.028***
Na^+^ mmol/L	136.23±0.92	134.12±1.21	127.26±6.91	136.74±1.68	*H(3)=6.576; p=0.087*
Fe umol/L	6.86±0.67	8.65±1.61	5.35±1.07^*^	2.43±0.22^*** ### £££^	*H(3)=20.818; **p=0.001***
Ferritin ug/L	470.35±150.76	752.92±124.56^*^	1429.63±255.27^***^	1340.8±260.25^***^	*H(3)=20.480; **p=0.001***
IL-6 pg/mL	117.45±8.80	142.27±23.34	241.44±97.31	330.25±63.99^*** ### £££^	*H(3)=25.193; **p=0.001***
MMP-9 ng/mL	401.05±388.19	933.05±826.10	911.54±608.69^*^	790.67±760.59	*H(3)=10.275; **p=0.016***
MMP-8 ng/mL	37.04±38.99	115.74±103.10	95.57±74.59^*^	75.95±84.25	*H(3)=11.358; **p=0.010***
BDNF ng/mL	0.80±0.51	0.67±0.27	0.56±0.24	0.62±0.23	*H(3)=2.380; p=0.497*
VEGF A ng/mL	2.15±1.93	3.70±3.65	5.50±3.54^*^	3.88±3.21	*H(3)=12.117; **p=0.007***

Kruskal-Wallis test was performed (H(df)= Kruskal-Wallis test statistics); followed by post-hoc test using the Mann-Whitney U test represented as ^*^ p<0.05; ^**^ p < 0.01; ^***^ p < 0.001 statistical significance compared to I group; ^#^ p<0.05; ^##^ p < 0.01; ^###^ p < 0.001 statistical significance compared to II group; ^£^ p<0.05; ^££^ p < 0.01; ^£££^ p < 0.001 statistical significance compared to III group.

**Table 3:** Spearman's correlation analysis between MMP-9, MMP-8, BDNF and VEGF A and cytokines or cytokines ratios in COVID-19 patients

	MMP-9	MMP-8	BDNF	VEGF A
	Rho (ρ)	Rho (ρ)	Rho (ρ)	Rho (ρ)
MMP-9	---	0.925^**^	0.708^**^	0.708^**^
MMP-8	0.925^**^	---	0.727^**^	0.727^**^
BDNF	0.535^**^	0.441^**^	---	0.564^**^
VEGF A	0.708^**^	0.727^**^	0.564^**^	---
IL-1β	0.094	0.090	-0.101	0.186
IL-6	0.123	0.145	-0.140	0.0001
IL-10	-0.177	-0.228	-0.279*	-0.384^**^
IL-17	0.176	0.174	0.245	0.281^**^
TNF-α	0.255	0.119	0.248	0.276^*^
TGF-β	0.435^**^	0.385^**^	0.510^**^	0.330^*^
IL-10 / IL-1	-0.221	-0.247	-0.445^**^	-0.200
IL-10 / IL-6	-0.242	-0.304^*^	-0.445^**^	-0.276^*^
IL-10 / IL-17	-0.354^**^	-0.411^**^	-0.411^**^	-0.452^**^
IL-10 / TNF-α	-0.248	-0.352^*^	-0.547^*^	-0.300

**Table 4:** Pearson′s correlation between serum MMP-9/BDNF ratio and biochemical parameters in COVID-19 patients related to disease severity I (mild), II (moderate), III (severe), and IV (critical)

MMP-9/BDNF ratio to	I (mild)	II (moderate)	III (severe)	IV (critical)
	Rho (ρ)	Rho (ρ)	Rho (ρ)	Rho (ρ)
White blood cell count	-0.042	0.157	0.006	0.203
Neutrophil count %	-0.072	0.213	-0.119	0.252
Lymphocyte count %	0.043	-0.317	0.090	0.028
Monocyte count %	0.074	0.152	0.122	-0.302
CRP	-0.003	0.160	0.095	0.283
D-dimer	-0.054	0.115	0.234	0.204
PCT	-0.047	-0.538	-0.219	0.374
CK	-0.016	0.105	-0.204	-0.238
LDH	-0.117	-0.335	-0.030	-0.283
Ferritin	-0.163	-0.251	0.184	-0.244
IL-6	0.159	0.413	0.280	-0.091
IL10 / IL6	-0.128	-0.431	0.033	-0.324
IL10 / IL17	-0.087	-0.569^*^	-0.101	-0.349
VEGF	0.062	0.504	-0.340	0.029
MMP-8	0.818^***^	0.944^***^	0.433	0.865^***^
