# Supplementary material for: MMP-9/BDNF ratio predicts more severe COVID-19 outcomes
Source: Int J Med Sci. 2022 Oct 24;19(13):1903–11. doi: 10.7150/ijms.75337 (PMC9682503; doi:10.7150/ijms.75337)
Supplement: Supplementary file 1 — Supplementary table. [file ijmsv19p1903s1.pdf]

	I (mild) n=22	II (moderate) n=16	III (severe) n=20	IV (critical) n=19
IL-1	161.25±109.18	79.74±22.91	170.21±47.85	117.19±33.78
TNF-α	58.76±35.57	40.25±16.80	108.7±47.06	53.46±16.29
IL-4	46.44±14.56	35.63±6.04	32.28±3.34	41.75±4.09
IL-10	118.78±13.71	143.23±56.20	172.64±85.63	380.44±103.43
IL-1/IL-4	2.35±0.64	3.19±1.02	6.28±1.66	3.31±0.95
IL-1/IL-10	1.04±0.55	0.90±0.53	0.20±0.12	0.09±0.02*
IL-6/IL-4	3.78±0.46	4.77±0.71	10.04±5.15	8.96±1.78*#
IL-6/IL-10	1.34±0.30	3.23±1.60	1.08±0.47	1.01±0.28
TNF-α/IL-4	0.97±0.31	0.97±0.43	3.57±1.37	1.16±0.34
TNF-α/IL-10	0.39±0.23	0.10±0.05	2.11±1.53	0.09±0.03

*p< 0.01 statistical significance compared to I group; # p< 0.05 statistical significance compared to II group
